# Ultrasound‐Based Skeletal Muscle Assessment for Prognostication in Cirrhosis: A Systematic Review

**DOI:** 10.1111/liv.70891

**Published:** 2026-09-18

**Authors:** Simone Di Cola, Paolo Gallo, Jakub Gazda, Saniya Khan, Lucia Lapenna, Stefania Gioia, Silvia Nardelli, Umberto Vespasiani‐Gentilucci, Manuela Merli

**Affiliations:** ^1^ Department of Translational and Precision Medicine Sapienza University of Rome Rome Italy; ^2^ Clinical Medicine and Hepatology Unit Fondazione Policlinico Universitario Campus Bio‐Medico Roma Italy; ^3^ 2nd Department of Internal Medicine Pavol Jozef Safarik University and L Pasteur Košice Slovakia; ^4^ Research Unit of Clinical Medicine and Hepatology, Department of Medicine and Surgery Università Campus Bio‐Medico di Roma Roma Italy

**Keywords:** cirrhosis, mortality, prognosis, sarcopenia, ultrasound

## Abstract

**Background and Aims:**

Sarcopenia is a major determinant of adverse outcomes in cirrhosis, yet CT‐ and MRI‐based assessments are difficult to implement in routine and longitudinal practice. Ultrasound is a feasible bedside alternative, but its prognostic role remains unclear. We performed a systematic review to evaluate the association between ultrasound‐derived skeletal muscle measures and mortality in adults with cirrhosis.

**Methods:**

This review followed PRISMA guidelines and was registered in PROSPERO (CRD420251056162). MEDLINE, Scopus, Web of Science and the Cochrane Library were searched to October 2025. Prospective studies assessing ultrasound‐based muscle parameters and mortality in cirrhosis were included. Risk of bias was evaluated using QUIPS.

**Results:**

Six prospective studies (*n* = 731) were included. Heterogeneity in muscle sites, ultrasound protocols, indexation methods, sarcopenia definitions and outcomes precluded meta‐analysis. Lower ultrasound‐derived muscle measures were consistently associated with higher mortality. In acute decompensation, reduced rectus femoris area predicted 90‐day mortality (sHR 0.57; 95% CI 0.38–0.85). Rectus abdominis thickness and psoas indices were also associated with mortality. In hospitalized decompensated cirrhosis, ultrasound‐defined sarcopenia was strongly associated with 6‐month mortality (HR 6.37; 95% CI 3.15–12.87). In critically ill liver transplant candidates, higher serial rectus femoris muscle area measurements were associated with better survival. In advanced chronic liver disease, sarcopenia was linked to increased 1‐year mortality.

**Conclusions:**

In our systematic review, ultrasound‐based muscle measures demonstrated a broadly consistent association with mortality; although not yet fully validated, they represent a promising bedside prognostic tool. Standardization is needed before clinical implementation.

## Highlights

- Ultrasound (US) muscle assessment in cirrhosis shows a generally consistent association with mortality but is not yet validated as a prognostic tool.
- Bedside muscle US enables repeatable assessment across settings.
- Studies' heterogeneity prevents pooled estimates and shared cut‐offs.
- Standardization is needed before routine clinical implementation.

## Lay Summary

People with liver cirrhosis often lose muscle, which is associated with worse outcomes. We reviewed six prospective studies including 731 patients and found that lower muscle measurements obtained by ultrasound were generally associated with a higher risk of death. Muscle ultrasound may therefore offer a simple and repeatable way to help assess and monitor patients, although standardized methods and validated thresholds are needed before it can be routinely used in clinical practice.

## 1 Introduction

Liver cirrhosis is a chronic pro‐inflammatory disease associated with a poor prognosis, particularly when patients transition from a compensated to a decompensated state [1]. Several factors may contribute to an accelerated worsening of the clinical course. Among these, the loss of skeletal muscle mass and function, known as sarcopenia, represents a frequent complication of cirrhosis. Sarcopenia reflects the complex interplay of reduced nutrient intake, hypercatabolic metabolism, chronic inflammation, decreased physical activity and hormonal dysregulation. Sarcopenia has consistently been associated with poorer quality of life, higher rates of infections, hepatic decompensation, reduced transplant‐free survival and post‐transplant complications [2, 3].

Cross‐sectional imaging (particularly computed tomography [CT]) with quantification of skeletal muscle area at the third lumbar vertebra (L3) and calculation of the skeletal muscle index (SMI) is considered the gold standard for muscle mass assessment in patients with cirrhosis [4]. However, CT‐based assessment is not universally available, involves radiation exposure, may be performed at variable time points with respect to clinical deterioration and requires dedicated software and expertise, all of which limit routine use and serial assessment in everyday hepatology care.

Ultrasound (US) assessment of skeletal muscle represents an attractive alternative. US is inexpensive, widely available, radiation‐free and can be performed at the bedside, enabling repeated measurements and potentially allowing longitudinal assessment of muscle loss. In other clinical settings, including geriatrics and critical care, muscle US measurements (e.g., quadriceps and rectus femoris thickness or cross‐sectional area) have shown good validity and reliability [5]. Nevertheless, in cirrhosis, the evidence is fragmented, with studies using different anatomical sites, acquisition protocols, indexation methods and definitions of ultrasound‐defined sarcopenia [6, 7]. Moreover, the extent to which muscle US can provide prognostic information beyond established severity scores (e.g., MELD and Child‐Pugh) remains unclear.

Therefore, we performed a systematic review to summarize prospective observational evidence on the prognostic role of muscle US parameters in predicting mortality in adult patients with cirrhosis. A comparison of muscle US with other techniques for assessing muscle mass was not an objective of the study; however, relevant comparative data were reported descriptively when available.

## 2 Methods

This systematic review was conducted in accordance with the Preferred Reporting Items for Systematic Reviews and Meta‐Analyses (PRISMA) statement [8]. The review question was defined using the PICO framework. The protocol was registered in the PROSPERO database (ID: CRD420251056162). The Grading of Recommendations Assessment, Development and Evaluation (GRADE) system was adopted to verify the consistency of the results [9].

### 2.1 Search Strategy

The MEDLINE (PubMed), Scopus, Web of Science and Cochrane Library databases were searched from inception to October 2025. The search strategy combined controlled vocabulary (MeSH and Emtree terms) and free‐text keywords related *to liver cirrhosis, imaging modalities, muscle assessment and clinical outcomes*. No language or date restrictions were applied. Reference lists of included studies and relevant reviews were also screened to identify additional eligible articles (backward citation analysis).

The following search string was used: ((‘Liver Cirrhosis’[MeSH] OR cirrhosis OR cirrhotics) AND ((‘ultrasound’) AND (‘MELD’ OR ‘child‐pugh’ OR ‘MR’ OR ‘MRI’ OR ‘CT’ OR ‘DEXA’ OR ‘handgrip strength’)) AND (‘quadriceps’ OR ‘psoas’ OR ‘rectus abdominis’ OR ‘rectus femoris’) AND (‘survival’ OR ‘mortality’ OR ‘outcome’ OR ‘liver transplantation’)).

### 2.2 Eligibility Criteria

Studies were considered eligible if they included adult patients with liver cirrhosis diagnosed according to clinical, laboratory, imaging and/or histological criteria. Studies were required to report muscle measurements obtained by ultrasonography—such as muscle thickness, cross‐sectional area, or indices normalized for patient height. Inclusion criteria were as follows: prospective observational studies reporting on the prognostic value of muscle US in cohorts of confirmed patients with liver cirrhosis, whose primary outcome was defined as liver‐related or all‐cause mortality during the follow‐up period. Exclusion criteria were: absence of detailed US protocol description and muscle mass measurements, design other than prospective, interventional study aiming to modify or improve muscle mass and studies that were not published as full‐text articles.

### 2.3 Study Selection and Data Extraction

Two investigators independently screened titles and abstracts to identify potentially eligible studies (SK and SDC). A third investigator (PG) subsequently reviewed the shortlisted articles to verify the accuracy and consistency of the selection process. Articles were then assessed in detail for eligibility by PG. Data from the included studies were then extracted independently using a predefined data‐extraction form (SDC, PG). Extracted variables included study design and setting, recruitment period, sample size, patient demographics, aetiology and stage of cirrhosis, inclusion and exclusion criteria, muscle ultrasound site(s) and acquisition protocol, definition of ultrasound‐defined sarcopenia, follow‐up duration, outcomes (including mortality) and effect estimates and any additional methods used for muscle mass or function assessment (e.g., CT‐derived SMI, anthropometry, handgrip strength). The entire procedure was supervised by senior hepatologists (SN, UVG and MM), who provided methodological oversight and resolved any discrepancies.

Rayyan software was used to facilitate study screening, conflict resolution and organization of records.

### 2.4 Risk of Bias Assessment

Risk of bias was assessed at the study level using the Quality In Prognosis Studies (QUIPS) tool, which evaluates six domains: study participation, study attrition, prognostic factor measurement, outcome measurement, study confounding and statistical analysis and reporting [10]. Two reviewers (SDC and PG) performed the assessment independently and disagreements were resolved by consensus or with an independent reviewer (JG).

The certainty of evidence for mortality was assessed using GRADE guidance for prognostic factors. Prognostic cohort evidence was initially considered high certainty and was evaluated across risk of bias, inconsistency, indirectness, imprecision and publication bias; large effects and exposure‐response gradients were considered as possible upgrading factors. Because the included studies evaluated non‐equivalent muscle sites and metrics, the assessment addressed the review‐level association between lower ultrasound‐derived muscle measurements and mortality rather than any individual anatomical site, index, or cut‐off. Two reviewers (SDC and JG) independently made the judgements, with disagreements resolved by consensus. The rationale for each judgement is reported in Appendix S1.

### 2.5 Feasibility of Quantitative Synthesis

Quantitative synthesis was deemed inappropriate at both overall and subgroup levels. An overall meta‐analysis was precluded by distinct anatomical sites, non‐comparable scales and incompatible statistical estimands (cause‐specific vs. competing‐risk hazard ratios). Restricted subgroup pooling (e.g., thigh/quadriceps measures) was also unfeasible due to substantial clinical heterogeneity among patients' (ICU vs. outpatients), unharmonized timing/normalization protocols and differing follow‐up durations. Consequently, a structured qualitative synthesis was performed.

## 3 Results

### 3.1 Study Selection

**FIGURE 1:**
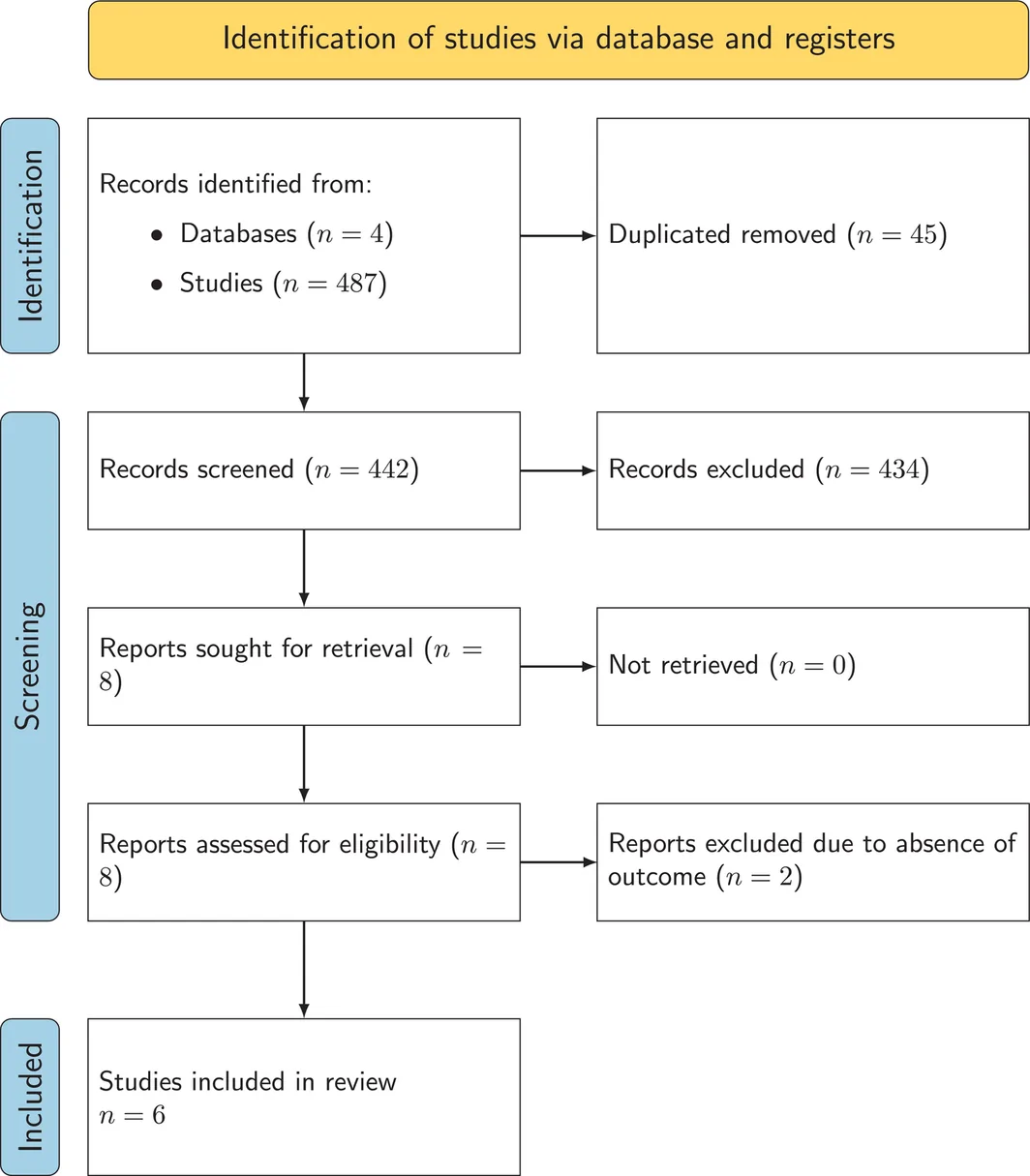
Flow diagram of study selection.

The database search and reference list screening yielded 487 records. After removal of 45 duplicates, 442 unique records were screened by title and abstract and 434 were excluded. Reasons for exclusion were not mutually exclusive. Specifically, 388 articles were excluded due to inadequate study design, 276 for involving a different study population, 248 for reporting outcomes not aligned with our objectives and 129 for an inappropriate publication type. Eight full‐text articles were assessed for eligibility. Two studies were excluded after full‐text review: one focused exclusively on the diagnostic correlation between ultrasound‐derived muscle measurements and other sarcopenia parameters, without evaluating clinical outcomes; the other did not report mortality as an outcome associated with ultrasound‐assessed muscle parameters. Six prospective observational studies were included in the descriptive synthesis (Figure 1).

### 3.2 Characteristics of Included Studies

The six included studies were published between 2019 and 2025 and enrolled a total of 731 participants. All studies were prospective cohorts. Most participants were men (approximately 62% to 84%) and reported mean/median age ranged from 49 to 64 years. Study populations and settings varied, as reported in Table 1.

**TABLE 1:** Characteristics of included studies and mortality findings.

Study (year)	Setting and population	*N*	Muscle US parameter	Follow‐up	Mortality findings
Gagliardi et al. (2025) [11]	Hospitalized acute decompensation; Phase 2 prospective cohort (Padua, Italy)	203	Right RF‐CSA (planimetry; mean of 3 measurements)	90 days	sHR 0.57 (95% CI 0.38–0.85) for 90‐day mortality (Fine–Grey; LT competing; adjusted for age, sex, MELD, infections)
Ciocîrlan et al. (2019) [12]	Cirrhosis cohort (in−/outpatients); tertiary centre (Romania)	61	Rectus abdominis thickness (mm; admission; minimal pressure)	Mean 11.9 months (2–28)	RA thickness: HR 0.701 (95% CI 0.533–0.922) for mortality (multivariable); psoas thickness not predictive
Hari et al. (2019) [13]	Decompensated cirrhosis; single‐centre cohort (reliable US in 54/75)	54	Psoas diameter indices (US‐PTHR mm/m; US‐PMI cm^2^/m^2^)	Median 12 months	Adjusted for MELD: US‐PTHR HR 0.825 (95% CI 0.701–0.973); US‐PMI HR 0.930 (95% CI 0.876–0.987)
Gödiker et al. (2024) [14]	Hospitalized advanced chronic liver disease with portal hypertension	63	Quadriceps thickness (proximal third) normalized to height (US‐SMI; cm/m); cut‐off 1.83 cm/m	12 months	Higher 1‐year mortality with US‐defined sarcopenia (27.8% vs. 3.7%, *p* = 0.013); 9 liver‐related deaths
Prakash et al. (2024) [15]	Hospitalized decompensated cirrhosis (India)	300	Quadriceps muscle index (cm/m^2^) + HGS; US‐defined sarcopenia (low strength + low thickness)	6 months	HR 6.37 (95% CI 3.15–12.87) for 6‐month mortality (sarcopenia vs. non‐sarcopenia)
Pita et al. (2020) [16]	ICU end‐stage liver disease awaiting liver transplantation (USA)	50	Serial bedside RFMA (planimetry; repeated every 48–72 h) indexed to BSA/height^2^	ICU stay; LT follow‐up median 17 months	RFMA associated with overall survival (adjusted HR 0.42, 95% CI 0.18–0.99; time‐dependent Cox)

Abbreviations: QMI, quadriceps muscle index; RA, rectus abdominis; RF‐CSA, rectus femoris cross‐sectional area; RFMA, rectus femoris muscle area; US, ultrasound; US‐PMI, ultrasound psoas muscle index; US‐PTHR, ultrasound psoas thickness‐to‐height ratio; US‐SMI, ultrasound‐derived skeletal muscle index.

### 3.3 Muscle Ultrasound Assessment

Considerable heterogeneity was observed in the anatomical sites and ultrasound metrics used. Two studies assessed thigh muscles using rectus femoris cross‐sectional area (including one with serial bedside measurements); two studies evaluated quadriceps muscle thickness and derived height‐indexed muscle indices; one study measured rectus abdominis thickness (with concurrent psoas thickness assessment); and one study assessed psoas diameter and derived ultrasound psoas indices. Ultrasound acquisition protocols differed with respect to transducer type and frequency, anatomical landmarks, number of measurements, use of probe pressure, whether measurements were averaged or maximal values were retained and whether parameters were normalized to height, height squared or body surface area (Table S1).

### 3.4 Included Studies and Main Findings

Gagliardi et al. [11] (Padua, Italy) reported a two‐phase prospective study in adults with cirrhosis. In Phase 1 (*n* = 77), patients underwent abdominal CT for liver nodule characterization or liver transplant assessment, allowing ultrasound measurements to be validated against CT‐derived muscle mass. In Phase 2 (*n* = 203), patients admitted with acute decompensation were enrolled for prognostic evaluation. The ultrasound protocol focused on bedside measurement of the right rectus femoris cross‐sectional area (RF‐CSA) using a planimetric technique: with the patient supine, the probe was kept perpendicular to the thigh's long axis at two‐thirds of the distance between the anterior superior iliac spine and the superior patellar border, using minimal pressure and abundant gel; three measurements were averaged and trained operators were blinded to CT results. In Phase 1, sarcopenia was defined by sex‐specific CT‐SMI thresholds and was present in 49% of participants; US‐RF‐CSA correlated strongly with CT‐SMI and showed excellent diagnostic performance (AUROC 0.90 in males and 0.92 in females) with sex‐specific RF‐CSA thresholds for optimal discrimination. In Phase 2, follow‐up continued until death, liver transplantation, or 90 days, whichever occurred first. The study reported 90‐day mortality and lower RF‐CSA was independently associated with a higher incidence of in‐hospital complications and with 90‐day mortality in competing‐risk models accounting for liver transplantation (reported as sHR).

Ciocîrlan et al. [12] (Bucharest, Romania) conducted a prospective cohort study in a tertiary centre including 61 patients with cirrhosis assessed at admission, during scheduled outpatient visits or hospital admissions for cirrhosis‐related complications. Ultrasound measurement targeted abdominal muscles: rectus abdominis (US‐RA) thickness was obtained in transverse section approximately 3 cm lateral to the umbilicus, applying minimal probe pressure; three measurements were taken on each side, the highest value per side was retained and the mean of the right and left US‐RA measurements was used for analyses. Psoas major thickness was also measured and nutritional/sarcopenia surrogates (handgrip strength, mid‐arm muscle circumference and subjective global assessment—SGA) were collected. Sarcopenia was not defined using CT/MRI criteria. Patients were followed for at least 6 months (mean follow‐up ~12 months) until transplant or death. During follow‐up, 18/61 patients died, all due to cirrhosis‐related complications. In multivariable analysis, lower US‐RA thickness independently predicted mortality (HR 0.701, 95% CI 0.533–0.922), whereas psoas thickness did not show prognostic value.

Hari et al. [13] (Slovenia and Switzerland) prospectively assessed ultrasound‐derived psoas indices in patients with decompensated cirrhosis. The study enrolled 75 consecutive patients, but a key feature was feasibility: only 54/75 (72%) had measurements considered reliable enough for downstream analyses. The ultrasound protocol involved identifying the psoas and recording the largest visible right psoas diameter in triplicate, using the mean value to derive an ultrasound psoas thickness‐to‐height ratio (US‐PTHR) and an ultrasound psoas muscle index (US‐PMI) (height‐squared normalization). The study did not define sarcopenia via CT cut‐offs; instead, prognostic associations were analysed using the continuous psoas‐derived indices. Follow‐up was approximately 12 months, during which mortality was recorded (alongside hospitalization due to further decompensation). Over this period, 15 patients died, predominantly from liver‐related causes. After adjustment including MELD, higher psoas indices were associated with lower mortality (US‐PTHR: HR 0.825, 95% CI 0.701–0.973; US‐PMI: HR 0.930, 95% CI 0.876–0.987).

Gödiker et al. [14] (Germany) described a monocentric prospective cohort enrolling 63 hospitalized patients with advanced chronic liver disease and portal hypertension. Quadriceps femoris thickness was measured within 48 h of admission by certified operators using a standardized protocol, with measurements collected at multiple locations and then normalized to height to generate an ultrasound‐derived skeletal muscle index (US‐SMI). A unisex cut‐off was derived to classify ‘ultrasound‐defined sarcopenia’, allowing identification of 36 of 63 patients as being sarcopenic (57%). Patients were followed for 1 year, with acute decompensation as the primary endpoint and liver‐related death as a key secondary endpoint. During follow‐up, 15/63 experienced acute decompensation and 9/63 died of liver‐related causes. Ultrasound‐defined sarcopenia was associated with a higher risk of acute decompensation (US: HR 16.6; *p* = 0.009; 95% CI 2.0–136.0) and with increased mortality compared with non‐sarcopenic patients (27.8% vs. 3.7%, *p* = 0.013).

Prakash et al. [15] (India) reported a prospective cohort study including 300 hospitalized patients with decompensated cirrhosis. Quadriceps muscle thickness was measured by ultrasound and expressed as a height‐indexed quadriceps muscle index (QMI; /height^2^), with the study detailing the technique (including probe compression to mitigate oedema‐related underestimation). Sarcopenia was defined by combining low handgrip strength with low US‐derived muscle thickness/QMI relative to a reference healthy population and the proportion classified as sarcopenic was 56%. Patients were followed for 6 months, with mortality as the outcome of interest. The study reported death events during follow‐up, including stratification by sarcopenia status and US‐defined sarcopenia was associated with a substantially higher risk of death (HR 6.37; 95% CI 3.15–12.87).

Pita et al. [16] (USA) conducted a prospective single‐centre cohort study enrolling 50 adults with end‐stage liver disease admitted to the ICU while undergoing evaluation for liver transplantation. The ultrasound protocol consisted of serial bedside measurements of right rectus femoris muscle area (RFMA), measured with US (US‐RFMA), using a linear‐array probe placed at three‐quarters of the distance from the anterior superior iliac spine to the superior patellar border, perpendicular to the thigh's long axis; three measurements were averaged and assessments were repeated every 48–72 h until transplant, death, or discharge. Analyses considered US‐RFMA as crude values and after normalization to body surface area and height^2^. The study focused on dynamic muscle loss rather than classifying baseline sarcopenia by a fixed threshold. Outcomes included survival to ICU discharge and overall survival (including follow‐up after transplantation). During the index ICU admission, 10 patients died or were discharged to hospice and additional deaths occurred after transplantation during follow‐up. In time‐dependent Cox models adjusted for sex and haemodialysis, higher US‐RFMA normalized to body surface area was associated with better overall survival (adjusted HR 0.42, 95% CI 0.18–0.99).

**FIGURE 2:**
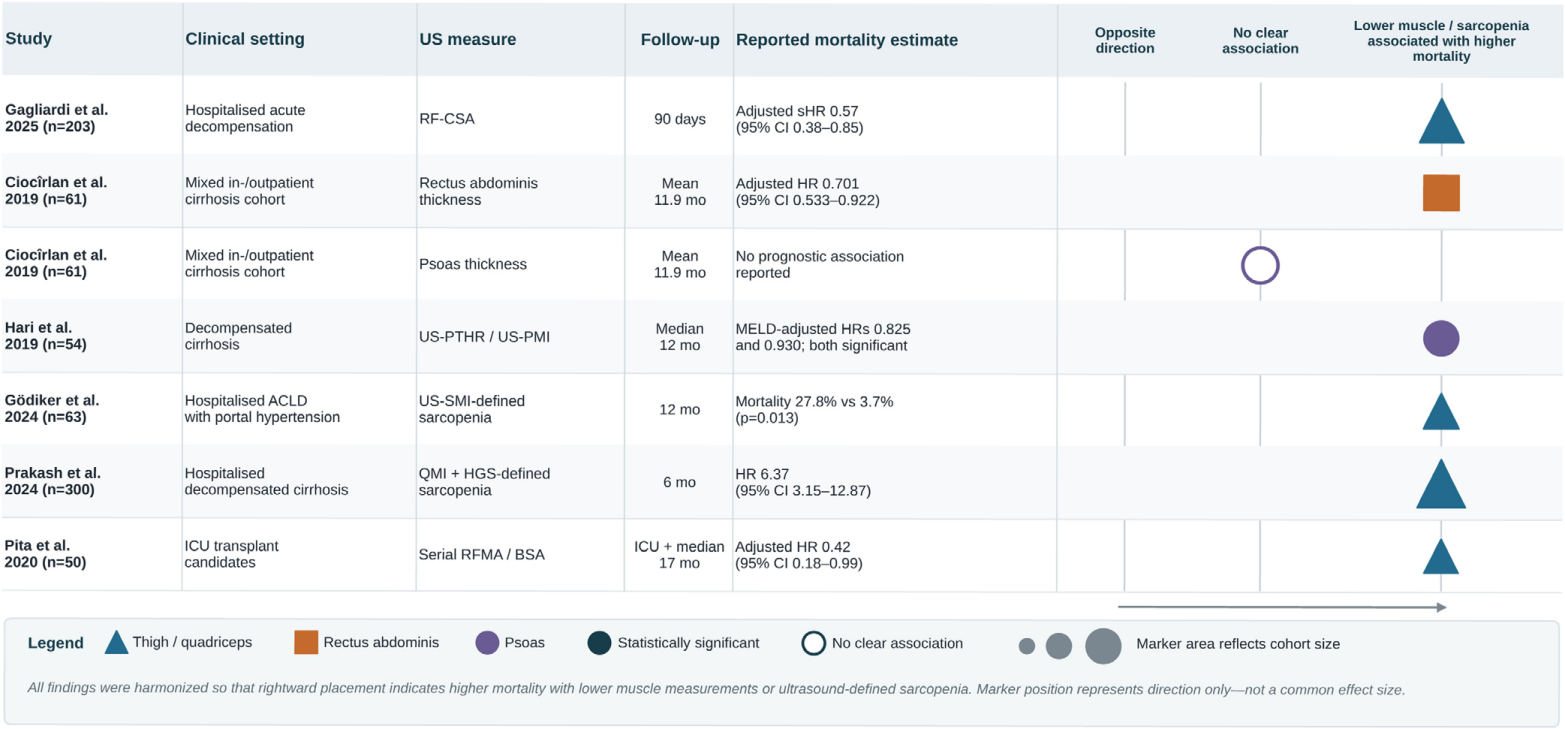
Effect‐direction plot on the directions of the mortality associations of ultrasound‐derived muscle measurements. Structured synthesis without pooling of the six cohorts, 731 participants.

As reported below, a quantitative pooling of the selected studies was unfeasible. We have then reported the findings in an effect direction plot (Figure 2) with a qualitative representation of the main findings.

### 3.5 Heterogeneity and Feasibility of Meta‐Analysis

Overall, there was substantial clinical and methodological heterogeneity across included studies, involving differences in patient populations (outpatients with decompensated cirrhosis, hospitalized acute decompensation cohorts, critically ill intensive‐care liver transplant candidates and advanced chronic liver disease with portal hypertension), US muscle sites and metrics (thickness, cross‐sectional area, serial versus single measurements and different indexation approaches), sarcopenia definitions and cut‐offs (sex‐specific versus unisex thresholds; CT‐anchored versus reference‐population based; continuous indices versus dichotomous classification), follow‐up durations and outcome definitions (90‐day mortality, 6‐month mortality, 1‐year liver‐related death and overall survival with liver transplantation treated as a competing event in some analyses) and the type and extent of confounder adjustment. These differences precluded meaningful quantitative pooling of effect estimates (Tables S2 and S3).

### 3.6 Risk of Bias

Risk of bias, assessed with the QUIPS tool, was overall low to moderate across the six included studies (Table 2). Study participation and outcome measurement were most frequently rated as low risk. The most common concerns related to study attrition (incomplete reporting of loss to follow‐up or censoring mechanisms) and study confounding (variable adjustment for established severity scores and clinical complications). Prognostic factor measurement was rated as moderate‐to‐high risk in some cohorts, reflecting heterogeneity in ultrasound protocols, the use of data‐driven cut‐offs and limited reporting of inter‐ and intra‐observer reproducibility in certain studies. The certainty of evidence for the association between lower ultrasound‐derived muscle measurements and mortality was rated as low (GRADE system). The evidence was downgraded for serious risk of bias, mainly because of incomplete reporting of attrition and censoring, variable adjustment for confounding and concerns regarding measurement and study‐specific cut‐offs and for serious imprecision because only six studies were available, mortality events were limited in several cohorts, some estimates had wide or incompletely reported confidence intervals and no pooled estimate could be calculated. The evidence was not downgraded for inconsistency, as the direction of association was broadly consistent, or for indirectness relative to the broad review question. Publication bias could not be excluded and no upgrading criteria were applied (Appendix S1).

**TABLE 2:** Risk of bias assessment summary (QUIPS domains; consensus rating).

Study	Participation	Attrition	Prognostic factor measurement	Outcome measurement	Confounding	Statistical analysis
Gagliardi et al. (2025) [11]	Low	Moderate	Low	Low	Low	Low
Ciocîrlan et al. (2019) [12]	Low	Moderate	Moderate	Low	Moderate	Low
Hari et al. (2019) [13]	Low	Moderate	Moderate	Low	Moderate	Moderate
Gödiker et al. (2024) [14]	Low	Moderate	High	Low	Moderate	Moderate
Prakash et al. (2024) [15]	Low	Moderate	Low	Low	Moderate	Low
Pita et al. (2020) [16]	Moderate	Moderate	Low	Low	Moderate	Moderate

## 4 Discussion

In this systematic review we evaluated whether US‐based assessment of skeletal muscle was associated with mortality in adults with cirrhosis. Across six prospective observational studies [11, 12, 13, 14, 15, 16], US‐derived muscle parameters measured at different anatomical sites and expressed as absolute values or indexed metrics were mostly associated with survival, with lower muscle measurements identifying patients at higher risk of death or other adverse clinical outcomes. Although the evidence was too heterogeneous to support quantitative pooling or thresholds, the overall direction of findings suggests that bedside ultrasound can capture clinically relevant impairment of physiologic reserve in cirrhosis. However, the certainty of evidence was low. Moreover, the available evidence largely reflects ultrasound‐based surrogates of muscle quantity, rather than sarcopenia as a multidimensional construct integrating muscle mass, strength and function. Notably, the current evidence does not yet establish the optimal anatomical site for muscle assessment, the added value of longitudinal over single‐time‐point measurements, or the incremental prognostic contribution of muscle ultrasound beyond established liver severity scores such as Child‐Pugh or MELD.

Sarcopenia is a well‐recognized risk factor for liver disease progression and mortality, particularly when associated with impaired muscle quality, or myosteatosis [3, 17]. Although fat infiltration cannot currently be reliably detected using available US techniques, muscle volume can be readily assessed at the bedside by ultrasound. Within the emerging concept of a multidimensional approach to chronic diseases, the integration of nutritional assessment into patient screening and monitoring represents a unique opportunity [18]. To date, the use of muscle evaluation in clinical practice remains limited [19, 20], mainly because the available techniques are difficult to incorporate into routine care. From this perspective, performing US either during routine hepatocellular carcinoma surveillance in patients with cirrhosis or at the bedside in hospitalized patients may provide substantial added value. Moreover, compared with gold‐standard imaging methods such as CT and MRI, US offers the advantage of repeatability, allowing clinicians to monitor nutritional changes over time in patients undergoing treatments that may affect muscle health. Likewise, in life‐threatening conditions such as acute‐on‐chronic liver failure, frequent and dynamic ultrasound assessment of muscle mass may improve daily risk stratification and prognostic evaluation. Direct comparison with established body‐composition techniques was beyond the primary scope of this review. We purposely selected prognostic rather than diagnostic‐accuracy investigations and only a limited number included a concurrent reference technique. Accordingly, the present findings should not be interpreted as demonstrating diagnostic equivalence or interchangeability between ultrasound and CT, MRI, or DXA. Dedicated diagnostic‐accuracy and agreement studies using standardized protocols and prespecified thresholds remain necessary.

Thigh US measures, especially those incorporating cross‐sectional area, were associated with adverse outcomes and mortality over short follow‐up horizons (Figure 2). Other cohorts conducted in different clinical contexts—including advanced chronic liver disease with 1‐year follow‐up [12, 13, 14] and critically ill ICU candidates awaiting transplantation [16]—support two complementary concepts: US can reflect baseline muscle depletion and it can also track dynamic muscle loss over time, which may be prognostically informative in rapidly evolving clinical states. It can also track dynamic muscle loss over time, which may be prognostically informative in rapidly evolving clinical states.

Despite these promising signals, a meta‐analysis could not be performed because heterogeneity was substantial at multiple levels. Ultrasound acquisition protocols varied in ways likely to affect measurement comparability, including differences in transducer type and frequency, anatomical landmarks (e.g., two‐thirds vs. three‐quarters ASIS–patella distance for thigh measurements), the role of probe pressure (minimal pressure vs. deliberate compression to mitigate oedema), the number of repetitions and whether mean or maximal values were retained. In addition, fluid overload, a common feature in decompensated cirrhosis and critical illness, may influence US‐derived muscle thickness through interstitial oedema, potentially confounding the assessment of true muscle mass. While this effect is likely more pronounced in subcutaneous tissues, its impact on muscle measurements should not be overlooked, particularly in the absence of standardized protocols controlling for probe pressure and tissue compressibility. Moreover, studies differed in the muscle parameter assessed (thickness versus area) and in whether and how values were normalized (height, height squared, or body surface area). Importantly, the construct of ‘sarcopenia’ was operationalized inconsistently—ranging from continuous predictors to study‐specific cut‐offs, sometimes sex‐specific and sometimes unisex and occasionally combined with functional measures such as handgrip strength. Clinical contexts and endpoints were also heterogeneous, with follow‐up windows spanning 90 days to 12 months and with transplantation variably treated as censoring or competing risk. Finally, adjustment strategies differed across studies; this matters because muscle depletion correlates strongly with liver disease severity and systemic inflammation and inadequate confounding control could either inflate or attenuate associations. At present, the optimal anatomical site for muscle ultrasound in cirrhosis remains uncertain. The quadriceps and particularly the rectus femoris may represent the most practical candidate for standardization because of its superficial location, ease of identification, suitability for serial bedside measurements and limited direct interference from ascites. In addition, preliminary comparative data suggest better clinical applicability and diagnostic performance for quadriceps‐derived indices than for psoas measurements. However, the current evidence is indirect and no sufficiently powered head‐to‐head study has established the superiority of one anatomical site over another. Future studies should compare muscle sites within the same cohort using standardized landmarks, probe pressure, indexation methods, reference techniques and clinical endpoints. Stratification for different clinical settings, stage of disease (i.e., according to MELD score or Child‐Pugh) and main confounders like age, sex and fluid retention must be incorporated in comprehensive analysis to find a practical and reproducible prognostic toll. Dynamic changes of US muscle measurement could also represent a prognostic marker but this needs further prospective studies.

Before clinical translation, muscle US should demonstrate not only prognostic association but also reproducibility across operators, centres and clinical settings. Importantly, when assessed, intra‐ and inter‐observer agreement has generally been reported as acceptable to good, although these data remain limited and often derive from single‐centre studies with experienced operators [11].

From a clinical perspective, muscle ultrasound should currently be considered a promising risk marker rather than a validated diagnostic or prognostic tool. Its most plausible future role may be to complement, rather than replace, established clinical and imaging assessments by identifying impaired physiological reserve and enabling serial monitoring. However, the current evidence is insufficient to recommend a specific muscle site, acquisition protocol, indexation method, or cut‐off for routine clinical use. Whether this instrument will add prognostic information beyond MELD is a future objective of investigation.

An individual participant data meta‐analysis might have helped harmonize patient‐level covariates, but it would not have overcome the substantial heterogeneity in anatomical sites, ultrasound parameters, acquisition protocols, measurement scales, follow‐up periods and mortality definitions. Moreover, the limited number of studies for each specific ultrasound measure would have restricted meaningful subgroup analyses.

In summary, prospective studies suggest that lower ultrasound‐derived muscle measures are associated with higher mortality in cirrhosis across different settings. However, standardized acquisition protocols, harmonized definitions (including indexing and cut‐offs) and consistent endpoint reporting are needed before robust pooled estimates and clinically actionable thresholds can be established. The quadriceps, particularly the rectus femoris, appear to be the most practical candidate for future standardization, although its superiority over other sites has not been demonstrated. Serial assessment represents a potentially valuable application, but its additional prognostic role is currently supported by only one study. Similarly, whether muscle ultrasound provides clinically meaningful incremental prognostic information beyond MELD or Child‐Pugh score remains unresolved. Standardized multicenter, head‐to‐head and longitudinal studies are therefore required before ultrasound‐based muscle assessment can be incorporated into routine prognostic algorithms.

## Author Contributions

Simone Di Cola and Paolo Gallo conceived the work, performed the literature review and methodological research, drafted the manuscript, provided substantial intellectual contributions and critically revised the manuscript in its entirety; Jakub Gazda performed the statistical analyses and critically reviewed the manuscript. Saniya Khan contributed to the literature review and methodological research. Lucia Lapenna, Stefania Gioia, Silvia Nardelli, Umberto Vespasiani‐Gentilucci and Manuela Merli provided critical intellectual input and critically revised the manuscript.

## Funding

The authors have nothing to report.

## Conflicts of Interest

The authors declare no conflicts of interest.

## Supporting information


**Appendix S1:** GRADE‐style certainty assessment for the association between ultrasound‐derived muscle measures and mortality.

**Table S1:** Ultrasound acquisition protocol characteristics across included studies.
**Table S2:** Study‐level feasibility of quantitative synthesis for mortality outcomes.
**Table S3:** Harmonization of mortality definitions, follow‐up and handling of liver transplantation.

## Data Availability

Data sharing not applicable to this article as no datasets were generated or analysed during the current study.
