# Supplementary material for: Ultrasound‐Based Skeletal Muscle Assessment for Prognostication in Cirrhosis: A Systematic Review
Source: Liver Int. 2026 Sep 18;46(10):e70891. doi: 10.1111/liv.70891 (PMC13587263; doi:10.1111/liv.70891)
Supplement: Supplementary file 1 [file LIV-46-0-s001.docx]

**Supplementary Table 1. Ultrasound acquisition protocol characteristics across included studies.**

| **Study (metric)** | **Transducer (type/frequency)** | **Anatomical landmark / site** | **No. of measurements and summary statistic** | **Probe pressure** | **Normalisation** |
| --- | --- | --- | --- | --- | --- |
| **Gagliardi 2025 (RF-CSA) ^11^** | Linear 5–15 MHz; convex 2–5 MHz if needed | Right thigh; probe ⟂ long axis at 2/3 distance ASIS–superior patellar border | 3 measurements; planimetric tracing; mean value | Minimal pressure | Unindexed; also indexed to height² |
| **Ciocîrlan 2019 (RA thickness; PM thickness) ^12^** | RA: linear 7.5 MHz; PM: convex 3.5 MHz | RA: ~2 cm above umbilicus, ~3 cm lateral; PM: oblique scan slightly above iliac crest | 3 per side; RA: highest value retained per side then mean of sides; PM: maximal per side then mean of sides | RA: minimal pressure; PM: NR | None reported |
| **Hari 2019 (psoas diameter) ^13^** | High-frequency abdominal probe (frequency NR) | Right psoas; sagittal subphrenic view just above iliac crest; maximal visible diameter | Triplicate; mean value (reliability criteria applied) | NR | US-PTHR (diameter/height) and US-PMI (radius²/height²) |
| **Gödiker 2024 (quadriceps thickness) ^14^** | 2–10 MHz transducer | Quadriceps femoris; upper/mid/lower third | 3 measurements with and without pressure; mean values | With and without pressure (analysis prioritised no pressure) | Thickness standardised to height (cm/m; US-SMI) |
| **Prakash 2024 (AMI; QMI) ^15^** | Broadband linear array 5–7.5 MHz | Arm: midpoint acromion–olecranon; Thigh: midpoint patella–greater trochanter; probe ⟂ thigh | Repetitions NR; right+left thickness summed for indices | Compression applied to reduce oedema-related underestimation | Both indices expressed as (right+left)/height² |
| **Pita 2020 (RFMA) ^16^** | 5‑cm linear-array transducer (frequency NR) | Right thigh; anterior aspect; 3/4 distance ASIS–superior patella; probe ⟂ long axis | 3 measurements averaged; serial every 48–72 h | NR | Crude; also normalised to BSA and height² |

Abbreviations: AMI, arm muscle index; ASIS, anterior superior iliac spine; BSA, body surface area; NR, not reported; QMI, quadriceps muscle index; RA, rectus abdominis; RF-CSA, rectus femoris cross-sectional area; RFMA, rectus femoris muscle area; US, ultrasound.

**Supplementary Table 2. Study-level feasibility of quantitative synthesis for mortality outcomes.**

| **Study** | **US prognostic factor and scale** | **Mortality effect estimate** | **Adjustment variables** | **Follow-up** | **Censoring, LT handling and statistical method** | **Reason for exclusion from pooling** |
| --- | --- | --- | --- | --- | --- | --- |
| Gagliardi et al. (2025) [11] | Right RF-CSA; continuous area (cm²), mean of three measurements; modelled per 1-cm² increase. | Adjusted sHR 0.57 (95% CI 0.38–0.85); p=0.006. | Age, sex, MELD score and baseline infection. An alternative model replaced MELD with ACLF. | 90 days from hospital inclusion. | Fine-Gray subdistribution hazards. LT was a competing event; administrative end at day 90. Twelve patients were lost to follow-up; their exact censoring date was not further specified. | Only baseline RF-CSA study in acute decompensation and only sHR. The competing-risk estimand is not interchangeable with conventional Cox HRs; no second clinically comparable RF-CSA study. |
| Ciocîrlan et al. (2019) [12] | Mean bilateral rectus abdominis thickness; continuous (mm), using the highest of three measurements per side and then the mean of both sides; modelled per 1-mm increase. | Adjusted HR 0.701 (95% CI 0.533–0.922); p=0.011. Psoas thickness was not prognostic. | Rectus abdominis thickness and ascites remained in the final Cox model; candidate predictors included MELD and MELD-Na. | Mean 11.9±5.63 months (range 2–28); at least 6 months planned. | Cox proportional hazards. Patients were followed until LT, death or at least 6 months. One LT occurred; formal treatment of LT as censoring was not explicitly stated. | Unique abdominal site and millimetre scale, variable follow-up, and no comparable rectus-abdominis study. LT handling was not fully specified. |
| Hari et al. (2019) [13] | Right psoas-derived US-PTHR (mm/m) and US-PMI (cm²/m²); continuous indices. Reliable measurements were available in 54/75 patients. | US-PTHR: HR 0.825 (95% CI 0.701–0.973); p=0.022. US-PMI: HR 0.930 (95% CI 0.876–0.987); p=0.017. | MELD was included in the adjusted analysis; the remaining model covariates were not reported in the review extraction. | Approximately 12 months. | Cox-type time-to-event analysis. General censoring rules, LT handling and any competing-risk method were not reported. | Both psoas indices and their normalization formulae were unique to this study; 28% of enrolled patients lacked reliable measurements. No comparable psoas-US study used the same scale. |

**Supplementary Table 2 (continued).**

| **Study** | **US prognostic factor and scale** | **Mortality effect estimate** | **Adjustment variables** | **Follow-up** | **Censoring, LT handling and statistical method** | **Reason for exclusion from pooling** |
| --- | --- | --- | --- | --- | --- | --- |
| Gödiker et al. (2024) [14] | Binary US-defined sarcopenia: proximal quadriceps thickness/height <1.83 cm/m; data-derived unisex cut-off. | 1-year all-cause mortality: 27.8% vs 3.7%; p=0.013. No mortality HR or CI was reported. | No adjusted mortality model. Multivariable models including MELD or Child-Pugh score evaluated acute decompensation, not mortality. | 12 months. | Kaplan-Meier/log-rank survival comparison. Loss to follow-up was censored. LT handling was not reported; no competing-risk analysis. | No compatible mortality effect estimate; binary data-derived cut-off and unique quadriceps-thickness metric. Mortality was a secondary outcome. |
| Prakash et al. (2024) [15] | Binary composite sarcopenia: low handgrip strength plus low US-derived arm/quadriceps muscle index below the 5th percentile of 100 healthy controls. | Cox HR 6.37 (95% CI 3.15–12.87); p<0.001 for 6-month mortality. | Covariates included in the reported Cox model were not specified in the study information extracted for this review. | 6 months; in-hospital and 3-month mortality were also reported. | Cox survival analysis. Time origin, general censoring rules, LT handling and competing-risk methods were not reported. | Composite exposure combines US muscle quantity with strength and uses a study-specific healthy-control cut-off. It is not comparable with isolated continuous US measures; model and censoring details were incomplete. |
| Pita et al. (2020) [16] | Serial right RFMA/BSA (cm²/m²), repeated every 48–72 h; continuous time-dependent covariate. | Adjusted HR 0.42 (95% CI 0.18–0.99); p=0.047 for death/discharge to hospice in overall survival. | Sex and receipt of hemodialysis. | From ICU admission to last contact; LT recipients had median follow-up of 17 months (95% CI 15.1–19.0). | Time-dependent Cox model. For overall survival, death in ICU or after LT and discharge to hospice were events; last contact was censoring and LT was not censoring. For survival to ICU discharge, LT and discharge home/elsewhere were censored. | Serial time-varying exposure, ICU LT-candidate population, unique BSA index, composite death/hospice endpoint and inclusion of post-LT deaths; not comparable with baseline single-time measurements. |

Interpretation: HR or sHR <1 indicates lower mortality with a higher muscle measurement; HR >1 indicates higher mortality in the sarcopenic group. Effect estimates and scales are presented as originally reported and were not converted to a common metric.

Abbreviations: ACLF, acute-on-chronic liver failure; BSA, body surface area; CI, confidence interval; HR, hazard ratio; ICU, intensive care unit; LT, liver transplantation; MELD, Model for End-Stage Liver Disease; RF-CSA, rectus femoris cross-sectional area; RFMA, rectus femoris muscle area; sHR, subdistribution hazard ratio; US, ultrasound; US-PMI, ultrasound psoas muscle index; US-PTHR, ultrasound psoas thickness-to-height ratio.

**Supplementary Table 3. Harmonization of mortality definitions, follow-up and handling of liver transplantation.**

| **Study** | **Mortality outcome and definition** | **Time origin and follow-up window** | **Deaths/events reported** | **General censoring rules** | **Handling of liver transplantation** | **Competing-risk method** |
| --- | --- | --- | --- | --- | --- | --- |
| Gagliardi et al. (2025) [11] | All-cause mortality within 90 days of inclusion. | Hospital inclusion for acute decompensation to day 90. | 53/203 deaths; 18 LT; 12 lost to follow-up. | Administrative end at day 90; handling/date of censoring for the 12 patients lost to follow-up was not further specified. | LT was explicitly treated as a competing event for mortality. | Yes: Fine-Gray subdistribution hazard model. |
| Ciocîrlan et al. (2019) [12] | Death during follow-up; all 18 deaths were attributed to cirrhosis-related complications. A separate 6-month mortality analysis was also performed. | Baseline/admission assessment; mean 11.9±5.63 months (range 2–28), with at least 6 months planned. | 18/61 deaths. | Patients without subsequent visits were contacted by telephone. Exact right-censoring rules were not specified. | Follow-up was described as continuing until LT, death or at least 6 months. One patient underwent LT; formal analytic handling was not stated. | No competing-risk analysis reported. |
| Hari et al. (2019) [13] | All-cause mortality; deaths were predominantly liver-related according to the review extraction. | Baseline psoas-US assessment; approximately 12 months. | 15 deaths among 54 patients with reliable US measurements. | Not reported. | Not reported. | No competing-risk analysis reported. |
| Gödiker et al. (2024) [14] | All-cause 1-year mortality; liver-related death due to ACLF was a secondary outcome. | Hospital enrolment to 12 months. | 11/63 all-cause deaths; 9 were liver-related/ACLF deaths. | Loss to follow-up was censored. | Not reported. | No; Kaplan-Meier/log-rank methods were used for survival. |
| Prakash et al. (2024) [15] | Overall all-cause mortality at 6 months; in-hospital and 3-month mortality were also reported. | Hospitalization/baseline assessment to 6 months. | Number of deaths not reported in the abstract or manuscript extraction. | Not reported. | Not reported. | No competing-risk analysis reported. |
| Pita et al. (2020) [16] | Overall survival: composite of death (during ICU stay or after LT) or discharge to hospice. A separate survival-to-ICU-discharge endpoint was analysed. | ICU admission to death/hospice or last contact; median post-LT follow-up 17 months among recipients. | 10 ICU deaths/hospice discharges, 1 later pre-LT death and 5 post-LT deaths; 36 patients underwent LT. | For overall survival, patients alive were censored at last contact. For the ICU-discharge endpoint, LT and discharge home/other facility were censored. | LT was not a censoring event for overall survival; follow-up continued after LT and post-LT deaths counted. LT was censoring for the ICU-discharge endpoint. | No competing-risk analysis; time-dependent Cox models were used. |

Abbreviations: ACLF, acute-on-chronic liver failure; ICU, intensive care unit; LT, liver transplantation; US, ultrasound.

NR/not reported denotes information that was not explicitly available in the published report or in the study-level extraction; no assumptions were made about unreported censoring or transplantation procedures.
