# Supplementary material for: Ultrasound‐Based Skeletal Muscle Assessment for Prognostication in Cirrhosis: A Systematic Review
Source: Liver Int. 2026 Sep 18;46(10):e70891. doi: 10.1111/liv.70891 (PMC13587263; doi:10.1111/liv.70891)
Supplement: Supplementary file 2 [file LIV-46-0-s002.docx]

**Appendix 1. GRADE-style certainty assessment for the association between ultrasound-derived muscle measures and mortality.**

| **Review question** | In adults with cirrhosis, are lower ultrasound-derived skeletal muscle measures associated with subsequent mortality? |
| --- | --- |
| **Outcome** | All-cause or liver-related mortality; reported follow-up ranged from 90 days to approximately 17 months. |
| **Body of evidence** | Six prospective cohort studies including 731 participants. Quantitative pooling was not feasible; the direction of association was synthesized narratively. |
| **Starting certainty** | High, following GRADE guidance for prognostic-factor evidence. |
| **Overall certainty** | LOW (⊕⊕○○). Low-certainty evidence suggests an association, but the magnitude of effect and the performance of any specific ultrasound measure remain uncertain. |

| **GRADE domain** | **Judgment** | **Rationale** |
| --- | --- | --- |
| **Risk of bias** | **Serious −1 level** | All studies had at least moderate concerns regarding attrition or censoring. Confounder adjustment was variable, and several cohorts had additional concerns related to prognostic-factor measurement, data-derived thresholds, or statistical analysis. These limitations could materially affect the observed associations. |
| **Inconsistency** | **Not serious No downgrade** | The direction of effect was broadly consistent: lower muscle measurements or ultrasound-defined sarcopenia were associated with higher mortality. One psoas-thickness result was null, but no study reported a clear association in the opposite direction. Differences in magnitude could not be evaluated on a common scale. |
| **Indirectness** | **Not serious for the broad question No downgrade** | The included studies directly enrolled adults with cirrhosis, assessed skeletal muscle with ultrasound, and evaluated subsequent mortality. However, diversity in settings, sites, protocols, and thresholds limits applicability to any specific clinical implementation. |
| **Imprecision** | **Serious −1 level** | Only six studies were available, several cohorts had small numbers of deaths, confidence intervals were wide or incompletely reported, and no pooled estimate could be calculated. The direction of association is more certain than its magnitude. |
| **Publication bias** | **Possible but undetected No downgrade** | With only six mostly single-centre studies, publication bias and selective reporting cannot be excluded and a formal small-study assessment is not informative. There was insufficient direct evidence to justify an additional downgrade. |
| **Large effect** | **No upgrade** | A large association was reported in one study, but exposures and estimands were not comparable across studies and residual confounding remained plausible. |
| **Exposure-response gradient** | **No upgrade** | Several studies analysed continuous measures, but a consistent gradient could not be evaluated across a standardized muscle parameter and scale. |
